# Antiphospholipid immune complexes as thrombosis risk marker

**DOI:** 10.18632/oncotarget.26612

**Published:** 2019-01-25

**Authors:** José A. Martínez-Flores, Manuel Serrano, Antonio Serrano

**Affiliations:** Department of Immunology, Instituto de Investigación Hospital Universitario 12 de Octubre, Madrid, Spain

**Keywords:** antiphospholipid, B2GPI, thrombosis, thrombocytopenia, angiogenesis

Antiphospholipid syndrome (APS) is an autoimmune vascular disorder characterized by several clinical characteristics, typically recurrent thrombosis and gestational morbidity, in patients carriers of antiphospholipid autoantibodies (aPL).

Diagnosis of APS is based on both clinical and laboratory criteria established through an International consensus statement. The simultaneous presence of at least one clinical criterion and one laboratory criterion is necessary to diagnose an APS patient. The clinical criteria include the presence of thrombotic events and/or gestational morbidity diagnosed by objective methods. In addition, APS patients may also have other vascular signs and symptoms (such as thrombocytopenia, migraine or livedo reticularis) that are not included in the consensus criteria. The laboratory criteria include positivity of any of the following: lupus anticoagulant, anticardiolipin antibodies (aCL) and/or of anti- Beta-2 Glycoprotein I (B2GP1) [1].

B2GP1 is a plasma protein that can freely circulate in the blood or is bound to lipoproteins or phospholipids such as cardiolipin. B2GP1 is synthesized in the liver, heart and kidney and its functions include the regulation of coagulation, fibrinolysis and angiogenesis [2]. It can also be found in platelet membranes and on the surface of endothelial cells [3], offering a suitable target antigen for autoantibodies.

The aPL are both diagnostic and pathogenic autoantibodies. Antibodies associated with vascular pathology are mainly directed against B2GP1. Anti- B2GP1 antibodies involve an imbalance in the coagulation processes but also in angiogenesis (as antiangiogenic) especially in placental endothelium [4].

Presence of aPL is necessary, but not sufficient, to produce an APS event, an additional trigger being needed (such as activation of innate immunity due to surgery, or infection). Its pathogenic mechanisms are not well known although it is considered that one of the basic mechanisms would be the activation of signalling pathways, such as that regulated by mTORC, in vascular cells [3, 5].

The predictive value of the presence of aPL in asymptomatic patients for incidence of APS-events is low. Additional research is needed to find predictive biomarkers to identify patients at greatest risk for APS-related clinical events.

Recently, we described the presence of circulating immune complexes (CIC) of aPL bounded to B2GP1 (B2-CIC) in the blood of aPL carriers [6] and its presence has been associated with occurrence of acute thrombotic events [7].

The best studied B2-CICs are those formed by IgA isotype immunoglobulin. However, B2-CIC formed by immunoglobulins of IgG and IgM isotypes have also been described.

B2-CIC are predictive for acute APS-events. Patients positive for aPL of IgA isotype are only at risk of thrombosis if they are B2-CIC positive. Patients who are carrier of aPL who are B2-CIC negative have the same risk as the aPL-negative control-group). Therefore, B2- CIC can be considered a biomarker that explores a new pathophysiological pathway of aPL [8].

The presence of B2-CIC in plasma implies that a hard union of aPL to the membrane-B2GP1 located in endothelial cells and platelets would also be formed. Thus, B2-CIC could be a marker for detecting aPL-mediated activation of signaling pathways in vascular cells. More research is needed to test this hypothesis and to better understand the effect of B2-CIC on the endothelium and platelets.

How these autoantibodies are generated remains unknown. B2GP1 mRNA is regulated in a cell cycle-dependent manner, with very low expression in low cycling conditions and increasing levels in proliferating cells, such as those involved in endothelial repairment.

Angiogenic state is critical for the tumor progression. It has recently been described that anti- B2GP1 antibodies are anti-angiogenic, both in vitro and in vivo experimental models, and that they also affect human endometrial angiogenesis [4]. On the other hand, other studies suggest that IgG anti-B2GP1 antibodies promote tissue factor-dependent tumor angiogenesis.

The vast majority of cancers have dysregulation of the cell cycle that can affect the expression of B2GP1 levels. In this way, because of the controversy on the role of B2GP1 and anti-B2GP1 antibodies, the role of B2GP1 and the behavior of B2-CIC in angiogenesis models should be evaluated and may help to understand the physiopathology and contribute to a new target for tumor angiogenesis.
